# Perception of Antibiotic Prescribing and Resistance Among Hospital Physicians in Medina City, Saudi Arabia

**DOI:** 10.7759/cureus.33296

**Published:** 2023-01-03

**Authors:** Abdulmajeed A Al Harbi, Ahmed F Al-Ahmadi, Abdulwahab G Algamdi, Sami Al-Dubai

**Affiliations:** 1 Family Medicine, Primary Healthcare Center of King Abdullah Airbase, Jeddah, SAU; 2 Preventive Medicine, General Directorate of Health Affairs of Medina, Ministry of Health, Madinah, SAU; 3 Family Medicine, Ministry of Health, Medina, SAU; 4 Preventive Medicine, Ministry of Health, Medina, SAU

**Keywords:** antibiotic administration, physicians, antibiotic prescription, perception, antibiotic resistance

## Abstract

Background

Overuse or misuse of broad-spectrum antibiotics appeared to be a major cause of increased antibiotic resistance. This study aimed to explore awareness and knowledge of antibiotic prescribing and resistance among hospital physicians in Medina, Saudi Arabia.

Methodology

A cross-sectional study was conducted among 223 physicians in seven public hospitals in Medina, Saudi Arabia. A validated self-administered questionnaire was used, including questions on sociodemographics, awareness about the current scope of antibiotic resistance, knowledge and frequency of antibiotic prescribing, confidence and input seeking, factors influencing antibiotic prescribing, and attitude toward antibiotic use. t-Test and analysis of variance (ANOVA) tests were used to compare total knowledge scores across the sociodemographic variables. The level of significance was set at 0.05.

Results

Most participants were aware that antibiotic resistance is a problem in Saudi Arabia (87.4%) and worldwide (93.3%). The majority of physicians (77.6%) were classified as having moderate knowledge, the mean knowledge score on antibiotics was 4.41, and 26.5% of the respondents prescribed antibiotics more than once daily. Most physicians (91.4%) were confident in antibiotic prescribing, and 94.1% of them consulted (at least *sometimes*) colleagues before prescribing. Factors associated with knowledge were age (*P *= 0.001), educational level (*P *= 0.002), working years (*P *= 0.004), current position (*P *= 0.003), specialty (*P *= 0.023), duration since graduation from medical school (*P *= 0.002), and duration since the last qualification (*P *= 0.022).

Conclusions

The majority of physicians had a moderate knowledge level of antibiotics, and most of them were prescribing antibiotics more than two times per week. Most respondents agreed that antimicrobial resistance is a worldwide concern and that it is a problem in Saudi Arabia. This study recommends training and courses on the fact that appropriate antibiotic prescribing should be ensured to have the best practice in antibiotic prescription among physicians.

## Introduction

Globally, antibiotic resistance is one of the greatest public health threats of the twenty-first century as a result of antibiotic overuse [1-2]. In 2017, the Centers for Disease Control and Prevention estimated up to 23,000 deaths in the United States per year and infection of 2 million people with antibiotic-resistant bacteria, resulting in $20 billion in excess direct health costs. The cost of treating antibiotic-resistant infections worldwide is estimated to be many billions of dollars per year [3]. In developing countries, including Saudi Arabia, antibiotic resistance has been rising, which contributes to increasing hospital-acquired infections that lead to high in-hospital mortality rates [4,5].

The consequences of antibiotic resistance affect both the economic sector such as increased treatment costs and lack of resources and the health of the population such as increased morbidity and mortality and decreased quality of life [6,7]. An effective change in physicians’ behaviors should be explored to develop an optimal intervention strategy. This requires understanding physicians’ knowledge regarding antibiotic use, resistance, factors influencing their decisions, and their actual practices [8].

Most of the previous surveys were conducted in Saudi Arabia to assess the general population's knowledge, attitude, and practice of antibiotic use and misuse, especially in upper respiratory tract infections [9,10]. Physicians have a major role in combating antibiotic resistance, and their knowledge and awareness are crucial for educating patients and controlling the overuse or misuse of antibiotics. Therefore, we aimed to assess awareness and knowledge of antibiotic prescribing and resistance among hospital physicians.

## Materials and methods

This cross-sectional study was conducted in seven governmental hospitals in Medina (Maternal and Children Hospital, King Fahad Hospital, Al-Ansar Hospital, Ohud Hospital, Rehabilitation Hospital, Al-Miqat Hospital, and Al Amal Psychiatric Hospital). All are teaching hospitals, with a total capacity of all hospitals around 1,000 beds [11]. The sample size was calculated using the Epi-Info 7 software (Centers for Disease Control and Prevention, Atlanta, GA, USA), considering a total population of 681 physicians and a 95% of confidence level, so the minimum required number was determined to be 246.

We included general practitioners, residents, specialists, consultants, and fellow physicians from five specialties: medicine, pediatrics, surgery, emergency, obstetrics, and gynecology. Physicians working in other specialties were excluded as they do not routinely prescribe an antibiotic. A validated self-administered questionnaire was distributed on-site during working hours. The questionnaire was adopted from previous studies in the United States, Malaysia, Peru, Egypt, and Pakistan [12-16]. Before release, it was reviewed by a panel of experts and was piloted among 10 physicians to assess the relevance and wording of the questions.

The 30-item questionnaire addressed the demographic and professional profile of the participants (eight questions), frequency of antimicrobial prescription (one question), basic knowledge (seven questions), awareness about the current scope of antimicrobial resistance (three questions), sources of information and continuing education about antimicrobial (one question), confidence and seeking inputs (two questions), factors influencing decisions around antimicrobial prescription (three questions), attitude toward antibiotic use (three questions), and the acceptability and appropriateness of potential interventions (one question).

Most of the questions used a five-point Likert scale, with answers ranging from *strongly disagree* to *strongly agree* or *never* to *always* or *useful* to *not useful*. The basic knowledge questions of the clinical indications, spectrum, administration, and pharmacology of antibiotics were as follows: three case-based questions that addressed the choice of antibiotics for treating acute diarrhea, upper respiratory tract infection, and sepsis in a patient with impaired renal function; one question addressed the safety of antibiotics during pregnancy; and three questions addressed the spectrum of antibiotics and ability to cross the blood-brain barrier [12-16]. Ethical approval was obtained from the ethical committee of the General Directorate of Health Affairs in Medina. Informed consent was obtained from the participants and confidentiality was assured, and the participants were given the right to withdraw from the study at any time.

Analysis was performed by using the Statistical Package for the Social Sciences (version 25.0, IBM, Armonk, NY, USA). Descriptive statistics were used to obtain the frequency and percentage for categorical variables and mean and standard deviation (SD) for continuous variables. For knowledge assessment, each correct response was scored 1, while a wrong response was scored 0 and then the sum of the scores was added to obtain the total knowledge score. Then the total knowledge score was categorized into three types (low, moderate, and high), with *low* representing two or fewer correct answers, *moderate* representing three to five correct answers, and *high* representing six or more correct answers. The t-test and ANOVA tests were used to compare the total knowledge scores across the sociodemographic variables. The level of significance was set at 0.05.

## Results

A total of 223 out of 246 physicians completed the questionnaire with a 91% response rate. The majority were males (60.5%), aged 25-35 years (63.7%), and practiced for six years or more (46.2%). More than half (52.5%) were general practitioners or residents, 28.2% were specialists, and 19.3% were consultants (Table 1).

**Table 1:** Demographic and professional profiles of the participants (n = 223). MBBS, Bachelor of Medicine, Bachelor of Surgery; PhD, Doctor of Philosophy

Characteristics	*n* (%)
Gender	
Male	135 (60.5)
Female	88 (39.5)
Age group (years)	
25-35	142 (63.7)
36-45	36 (16.1)
>45	45 (20.2)
Time since graduation from medical school (years)	
<10	130 (58.3)
10-30	89 (40.0)
>30 years	4 (1.7)
Educational level	
Bachelor (MBBS)	137 (61.5)
Master or diploma	33 (14.8)
Board or PhD	53 (23.7)
Duration since the highest qualification (years)	
<10	172 (77.1)
10-30	48 (21.5)
>30	3 (1.4)
Working experience (years)	
≤2	67 (30.0)
3-5	53 (23.8)
>5	103 (46.2)
Department	
Medicine	76 (34.1)
Pediatrics	40 (17.9)
Surgery	38 (17.1)
Emergency	36 (16.1)
Obstetrics and gynecology	33 (14.8)
Current position	
General practitioner/resident	117 (52.5)
Specialist	63 (28.2)
Consultant	43 (19.3)

The mean (±SD) knowledge score was 4.41 (±1.1) with a range of 4-7. According to the knowledge level score, the majority of physicians (77.6%) were classified as moderate knowledge, 16.1% were classified as high knowledge, and 6.3% were classified as low knowledge.

In univariate analysis, factors associated significantly with knowledge were age group (*P *= 0.001), educational level (*P *= 0.002), working experience (*P *= 0.004), current position (*P *= 0.003), department (*P *= 0.023), time since graduation from medical school (*P *= 0.002), and duration since the highest qualification (*P *= 0.022) (Table 2).

**Table 2:** Association between the total knowledge score and sociodemographic characteristics of the participants (n = 223). MBBS, Bachelor of Medicine, Bachelor of Surgery; MD, Doctor of Medicine; PhD, Doctor of Philosophy; SD, standard deviation

	Mean	SD	P-value
Gender			
Male	2.13	0.45	0.330
Female	2.04	0.47	
Age (years)			
25-35	2.02	0.45	0.001
36-45	2.13	0.42	
>45	2.31	0.46	
Educational level			
Bachelor (MBBS) or MD	2.02	0.44	0.002
Master or diploma	2.12	0.48	
Board or PhD	2.28	0.45	
Working years			
≤2	1.97	0.49	0.004
3-5	2.05	0.45	
>5	2.20	0.42	
Current position			
General practitioner/resident	2.00	0.45	0.003
Specialist	2.19	0.47	
Consultant	2.23	0.42	
Department			
Medicine	2.13	0.52	0.023
Pediatrics	2.27	0.50	
Surgery	2.05	0.40	
Emergency	1.09	0.41	
Obstetrics and gynecology	2.03	0.30	
Time since graduation from medical school			
< 10 years	2.01	0.46	0.002
10-30 years	2.17	0.41	
> 30 years	2.33	0.48	
Duration since highest qualification (years)			
<10	2.05	0.46	0.022
10-30	2.25	0.43	
>30	2.33	0.57	

Table 3 shows that the mean knowledge scores of correct answers for each question ranged from 4.5% (item 3) to 94.6% (item 4).

**Table 3:** Mean knowledge scores of correct answers (n = 223).

Question and possible answers (correct answer in brackets)	Correct answer, *n* (%)
A 40-year-old woman has had diarrhea for four days (three stools per day). She had no fever during the examination or last few days. Which treatment do you propose?	
a) Ciprofloxacin	
b) Trimethoprim-sulfamethoxazole	
c) No antibiotic treatment, only oral rehydration (Correct)	201 (90.1)
A 32-year-old male went to the clinic complaining of fever (39 °C), nasal discharge, and throat pain for three days. Which antibiotic will you recommend?	
a) Amoxicillin	
b) Clarithromycin	
c) Trimethoprim-sulfamethoxazole	
d) No need for antibiotic use (Correct)	101 (45.3)
You have two cases of impaired kidney function. Case A: a 68-year-old man with cellulitis on clindamycin; case B: a 64-year-old woman with diabetes and sepsis on ceftriaxone and gentamicin. In which case would you need to adjust the antibiotic dose?	
a) Case A	
b) Case B	
c) Both cases A and B	
d) Neither case A or B (Correct)	10 (4.5)
Which one of the following antibiotics is safe during pregnancy?	
a) Amoxicillin (Correct)	211 (94.6)
b) Ciprofloxacin	
c) Gentamicin	
Which one of the following antibiotics has the best activity against anaerobes?	
a) Ciprofloxacin	
b) Metronidazole (Correct)	198 (88.8)
c) Trimethoprim-sulfamethoxazole	
Methicillin-resistant Staphylococcus aureus is susceptible to:	
a) Cephalothin	
b) Cefuroxime	
c) Ceftriaxone	
d) None of the above (Correct)	168 (75.3)
Which one of the following antibiotics most effectively crosses the blood-brain barrier?	
a) Clindamycin	
b) Ceftriaxone (Correct)	95 (42.6)
c) Vancomycin	

Regarding awareness about the current scope of antibiotic resistance, 208 (93.3%) of the respondents agreed that antimicrobial resistance is a worldwide concern and 87.4% agreed that antimicrobial resistance is a problem in Saudi Arabia, while 62.3% agreed that it is a problem in their daily practice. The majority agreed upon the perception of overuse of antibiotics in both Saudi Arabia hospitals (86.1%) and the community (85.6%) (Table 4).

**Table 4:** Awareness of the scope of antimicrobial resistance (n = 223). AMR, antimicrobial resistance; AM, antimicrobial

	Strongly agree/agree, *n* (%)	Neutral, *n* (%)	Strongly disagree/disagree, *n* (%)
AMR is a problem worldwide.	208 (93.3)	12 (5.4)	3 (1.3)
AMR is a problem in my country.	195(87.4)	26 (11.6)	2 (1.0)
AMR is a problem in daily practice.	139 (62.3)	57 (25.5)	27 (12.2)
AM are overused in our hospitals.	192 (86.1)	27 (12.1)	4 (1.8)
AM are overused in our community.	191 (85.6)	26 (11.7)	6 (2.7)

Regarding confidence and input seeking, most respondents (91.4%) felt either *very confident* (43%) or at least *somewhat confident* (48.4%) about optimal antibiotic prescribing. Only 19 (8.5%) physicians felt hesitant about optimal antibiotic prescribing. The majority (94.1%) discuss their decision to prescribe antibiotics with their senior colleagues at least *sometimes,* while the remaining (5.8%) never seek any opinion from senior colleagues. Regarding the acceptability and appropriateness of potential intervention, most of the participants strongly agreed or just agreed with the development of antimicrobial prescribing educational programs (91.5%) and agreed that a local antimicrobial guideline would be more useful than an international one (78.2%).

Table 5 shows that 27 (32.3%) participants reported that they had not received any education as part of the academic activities within their departments last year, 113 (50.7%) had received one to three education sessions, and 7 (3.1%) had received more than 10 education sessions. Regarding external courses, 120 (53.8%) did not receive any external courses, 83 (37.2%) received one to three courses, and 2 (1%) received more than 10 courses.

**Table 5:** Frequency of receiving education on antibiotics during the last year (n = 223).

Frequency	Department activities, *n* (%)	External courses, *n* (%)
None	72 (32.3)	120 (53.8)
1-3 times	113 (50.7)	83 (37.2)
4-10 times	31 (13.9)	18 (8.0)
More than 10 times	7 (3.1)	2 (1.0)

A total of 59 (26.4%) participants prescribed antibiotics *more than once a day*, 53 (23.8%) prescribed antibiotics *three to five times per week*, 46 (20.6%) prescribed antibiotics *one to two times per week*, 45 (20.2%) prescribed it *less than once a week,* and 20 (9%) prescribe it *once a day*.

Overall, the most useful source of information reported by the participants was the internet (57%) followed by information from a senior colleague or fellow (47.5%) and information from the Ministry of Health Drug Formulary (37.2%). Hospital pharmacy and British National Formulary (BNF) were reported as useful sources of information by 35% and 29.1%, respectively, among participants. The least useful source of information was the Sanford Antimicrobial Guide (21.1%) (Table 6).

**Table 6:** Utility of various antimicrobial continuing education sources. BNF, British National Formulary; MOH, Ministry of Health

Educational source	Useful,* n* (%)	Neutral, *n* (%)	Not useful, *n* (%)	Not familiar, *n* (%)
Information from senior colleagues	106 (47.5)	90 (40.4)	14 (6.3)	13 (5.8)
BNF	65 (29.1)	62 (27.8)	22 (9.9)	74 (33.2)
Internet sources	127 (57.0)	62 (27.8)	27 (12.1)	7 (3.1)
Sanford antimicrobial	47 (21.1)	50 (22.4)	27 (12.1)	99 (44.4)
Pocket-based antimicrobial	75 (33.6)	58 (26.0)	28 (12.6)	62 (27.8)
MOH Drug Formulary	83 (37.2)	61 (27.4)	27 (12.1)	52 (23.3)
Hospital pharmacy	78 (35.0)	87 (39.0)	27 (12.1)	31 (13.9)

In this study, respondents were asked about the factors influencing their antibiotic prescribing. Around three-quarters (70.4%) of the participants *strongly agree* or *agree* that patients’ demand for antimicrobial therapy contributes to their overuse in the community, whereas only half (50.3%) did so in a hospital setting. More than third (36.8%) agreed with the statement that the antimicrobial available in their hospitals are of poor quality and are not effective. Nearly half of them (44.9%) agreed that the choice of antibiotic was affected by its availability in their setting more than by the cause of infection (Table 7).

**Table 7:** Perception of factors influencing the decision on antimicrobial prescription. AM, antimicrobial

	Strongly agree, *n* (%)	Agree, *n* (%)	Neutral, *n* (%)	Disagree, *n* (%)	Strongly disagree, *n* (%)
Patients’ demands for AM contribute to overuse in the community.	73 (32.7)	84 (37.7)	48 (21.5)	14 (6.3)	4 (1.8)
Patients’ demands for AM contribute to overuse in hospitals.	32 (14.3)	80 (36.0)	68 (30.5)	27 (12.1)	16 (7.1)
I suspect that AM available in my hospital is of poor quality and not effective.	27 (12.1)	55 (24.7)	70 (31.4)	59 (26.4)	12 (5.4)
My choice of AM is more affected by the availability than the cause of the infection.	24 (10.8)	76 (34.1)	48 (21.5)	54 (24.2)	21 (9.4)

## Discussion

This study aimed to explore awareness and knowledge of antibiotic prescribing and resistance among hospital physicians. In this study, the mean knowledge score was 4.41 ± 1.11, which was lower than two previous studies from Malaysia (5.31 ± 1.19) and Peru (6.0 ± 1.3), whereas higher than the study reported from Pakistan (3.66 ± 1.1) [12,14,15]. In this study, there was a significant association between years of working and knowledge compared to previous studies, which did not find such an association [14,16].

Our finding indicated that a longer duration of practice ensures good knowledge of antibiotic prescribing. Overall, the basic knowledge about the clinical indications, spectrum, administration, and pharmacology of antibiotics ranged from moderate to high. Despite this surprising score, it should be noted that more than half of the participants recommended using antibiotics for upper respiratory tract infections. Therefore, we highly encourage raising the study recommendation for a focused educational activity to higher authority at the Ministry of Health.

Regarding the confidence level, this study showed that only 43% of the respondents were very confident in choosing the right antibiotics, which is lower than the study reported from Peru among residents (47%) and attending physicians (82%), whereas it is higher than reported studies from the United States (21%), DR Congo (11.4%), and Malaysia (4%) [14-17].

 In this study, there was a significant association between years of working and confidence level scores. A similar finding was reported by a previous study from Peru [15]. However, two previous studies did not find a significant association [14,17]. Notably, most of the respondents in this study (94.1%) reviewed their decision with their senior colleagues when prescribing antibiotics at least *sometimes* compared to Pakistan (80%), DR Congo (79.4%), and Malaysia (77%) [12,14,17].

In our study, the respondents were very receptive to intervention in prescribing antibiotics. Most of the respondents would prefer national antibiotic guidelines more than international guidelines because they felt that the local data would be more useful for their practices. Therefore, national guidelines will certainly be more focused and useful for doctors who practice locally. The awareness of participants about antibiotic resistance is a worldwide as well as a national problem that was very high among the respondents in this study, which is consistent with the previous studies [12,15]. Most of the respondents in this study agreed that antibiotics were overused in the hospitals and by the community. This finding is consistent with the previous studies [15].

In this study, 26.5% of the participants have prescribed antibiotics *more than once a day* in the last week, which is lower than previous studies from Pakistan (66%), Egypt (60%), Malaysia (52%), DR Congo, and Peru (49%) [12-15,17]. In Scotland and France, 48% of the physicians prescribed antibiotics *two times or less* in the last week [18]. Regarding the factors that influence antibiotic prescribing in this study, two-thirds of the respondents in this study identified the patient's demand for an antibiotic as a key factor. A similar finding was reported by the previous studies [15,17].

In this study, the most common source of information on antibiotics was the internet. Similar results were found in Malaysia and Peru [14,15]. However, in the United States and Egypt, the most common source of information was *senior colleagues* [13,16]. In this study, antibiotics guidelines, such as BNF and the Sanford Antimicrobial Guide were considered the least important source of education. On the other hand, lectures and workshops were the most common way of education in Ethiopia, Scotland, and France (73%) [18,19]. In this study, 36.7% agreed with the statement that the antimicrobials available in their hospitals are of poor quality, which is lower than studies reported from Peru (57%) and Malaysia (79%) [14,15].

A potential limitation could be some of the participants may tend to answer with socially desirable responses. This potential bias is minimized by ensuring complete confidentiality regarding the finding of the survey. Another limitation of this study is that not all physicians in Medina have been included in the study; therefore, the results cannot be generalized to all the physicians in Medina.

## Conclusions

The majority of physicians had a moderate knowledge level of antibiotics, and most of them were prescribing antibiotics more than two times per week. Most respondents agreed that antimicrobial resistance is a worldwide concern, and it is a problem in Saudi Arabia. This study recommends that training and courses on appropriate antibiotic prescribing should be ensured to have the best practice in antibiotic prescription among physicians.
